# Supplementary material for: Faecal metabolites as a readout of habitual diet capture dietary interactions with the gut microbiome
Source: Nat Commun. 2025 Dec 4;16:10051. doi: 10.1038/s41467-025-66046-7 (PMC12678775; doi:10.1038/s41467-025-66046-7)
Supplement: Supplementary file 1 — Supplementary Information [file 41467_2025_66046_MOESM1_ESM.pdf]

SUPPLEMENTARY INFORMATION

Faecal metabolites as a readout of habitual diet capture dietary interactions with the gut microbiome.

Robert Pope [1], Alessia Visconti [1,2], Xinyuan Zhang [1], Panayiotis Louca [1,3], Andrei-Florin Baleanu [1], Yu Lin [1], Francesco Asnicar [4], Kate Bermingham [5,6], Kari E. Wong [7], Gregory A. Michelotti [7], Jonathan Wolf [6], Nicola Segata [4], Sarah E. Berry [5,6], Tim D. Spector [1,6], Emily R. Leeming [1], Rachel Gibson [5], Cristina Menni [1,8,9,+], Mario Falchi [1,+,*]

[1] Department of Twin Research & Genetic Epidemiology, King's College London, London, SE1 7EH, UK
[2] Centre for Biostatistics, Epidemiology, and Public Health, Department of Clinical and Biological Sciences, University of Turin, Turin, Italy
[3] Human Nutrition & Exercise Research Centre, Newcastle University, Newcastle, UK
[4] Department CIBIO, University of Trento, Trento, Italy.
[5] Department of Nutritional Sciences, King's College London, London, UK.
[6] Zoe Limited, London, UK.
[7] Metabolon, Research Triangle Park, Morrisville, NC, USA.
[8] Department of Pathophysiology and Transplantation, Università Degli Studi di Milano, Milan, Italy
[9] Fondazione IRCCS Cà Granda Ospedale Maggiore Policlinico, Angelo Bianchi Bonomi Hemophilia and Thrombosis Center, 20122 Milan, Italy.

[+] These authors jointly supervised this work: Mario Falchi & Cristina Menni

* Correspondence should be addressed to: Mario Falchi (mario.falchi@kcl.ac.uk)

Table of Contents

Supplementary Figures **3**
Supplementary Figure 1 3
Supplementary Figure 2 4
Supplementary Figure 3 5
Supplementary Figure 4 6
Supplementary Figure 5 7
Supplementary Figure 6 8

Supplementary Tables **9**
Supplementary Table 1 9
Supplementary Table 2 10
Supplementary Table 3 11
Supplementary Table 4 12
Supplementary Table 5 13

References **14**

Supplementary Figures

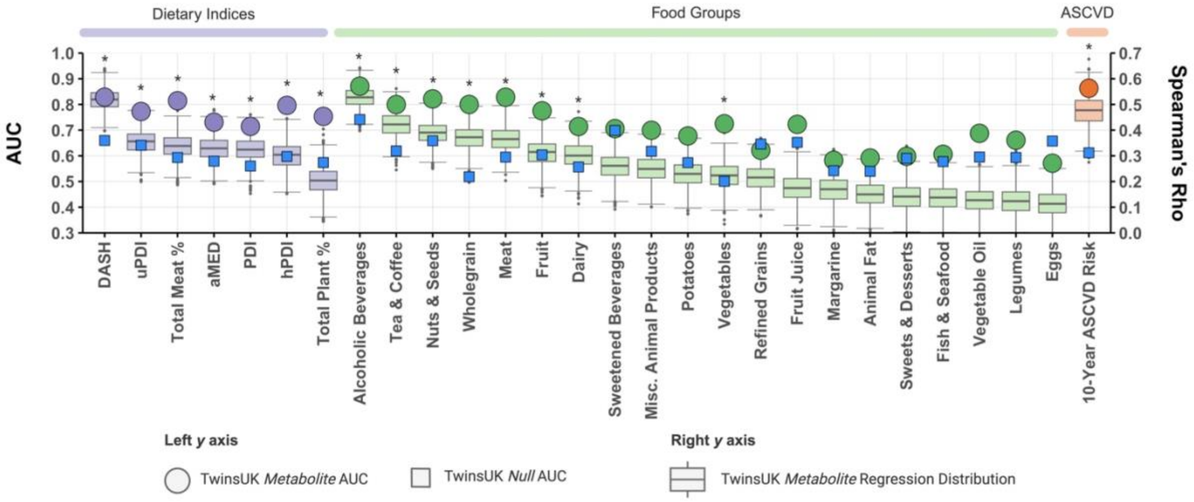

Supplementary Figure 1: Prediction of adherence to 7 dietary patterns, habitual intakes of 20 food and beverage groups and 10-year atherosclerotic cardiovascular disease (ASCVD) risk using faecal metabolites as predictors for RF binary classification (TwinsUK n=905) and regression models (TwinsUK n=1,810). The left axis reports the AUC values for the predictions made using the TwinsUK 20% hold out sets for the *null* (square) and *metabolite* (circle) models. The *null* models were trained solely on the covariates age, sex and BMI (only BMI for 10-year ASCVD risk) whereas the *metabolite* models included the covariates plus faecal metabolite data. Significant differences between the *null* and *metabolite* model AUC scores were determined by DeLong's test (* indicates significantly different AUC scores). The right y-axis reports the distribution of the Spearman's rank correlation coefficients between predicted and observed labels for the *metabolite* models using the TwinsUK 20% testing set, computed by 1,000 bootstrapped samples with replacement. Boxplots show the median (centre line) and interquartile range (box limits) with whiskers extending to 1.5 times the interquartile range. *Abbreviations: DASH, Dietary Approaches to Stop Hypertension; PDI, Plant-based Diet Index; hPDI, Healthful Plant-based Diet Index; uPDI, Unhealthful Plant-based Diet Index; aMED, Alternate Mediterranean Diet Score; ASCVD, 10-year atherosclerotic cardiovascular disease risk.*

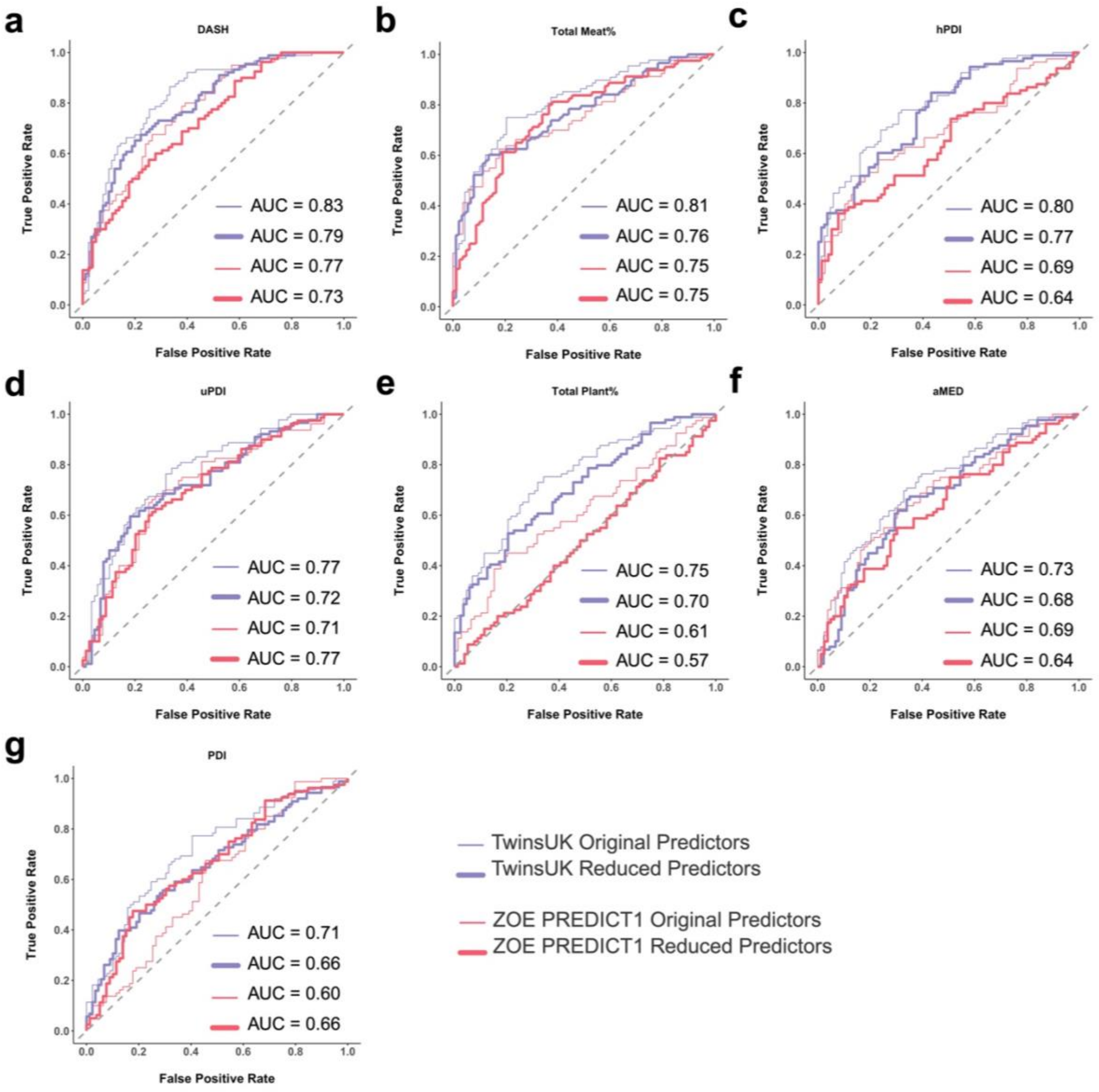

Supplementary Figure 2: Receiver operating characteristic (ROC) curves for models trained with faecal metabolites to predict adherence to dietary patterns (Purple: TwinsUK n=905; Red: ZOE PREDICT1 n=159). Models tested with the original input features used to train the models and the reduced subset of input features for both the TwinsUK and ZOE PREDICT1 cohorts. Area under the curve (AUC) is reported for dietary patterns defined by the: a, Dietary Approaches to Stop Hypertension (DASH); b, total meat %; c, Healthful Plant-based Diet Index (hPDI); d, Unhealthful Plant-based Diet Index (uPDI); e, total plant %; f, Alternate Mediterranean Diet Score (aMED); g, Plant-based Diet Index (PDI) indices.

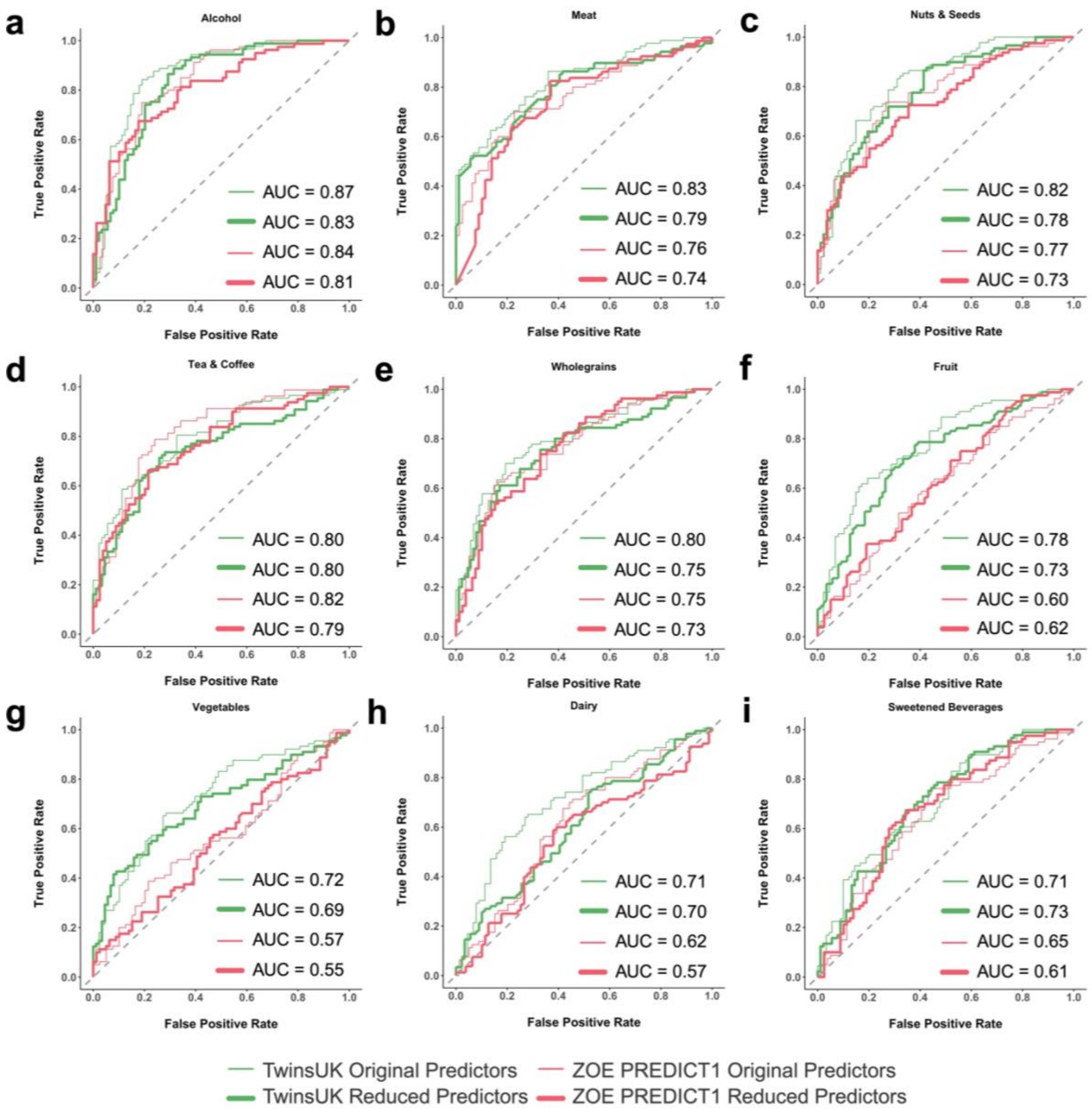

Supplementary Figure 3: Receiver operating characteristic (ROC) curves for models trained with faecal metabolites to predict habitual intakes of food & beverage groups (Green: TwinsUK n=905; Red: ZOE PREDICT1 n=159). Models tested with the original input features used to train the models and the reduced subset of input features for both the TwinsUK and ZOE PREDICT1 cohorts. Area under the curve (AUC) is reported for: **a**, alcohol; **b**, meat; **c**, nuts & seeds; **d**, tea & coffee; **e**, wholegrains; **f**, fruit; **g**, dairy; **h**, dairy; **i**, sweetened beverages.

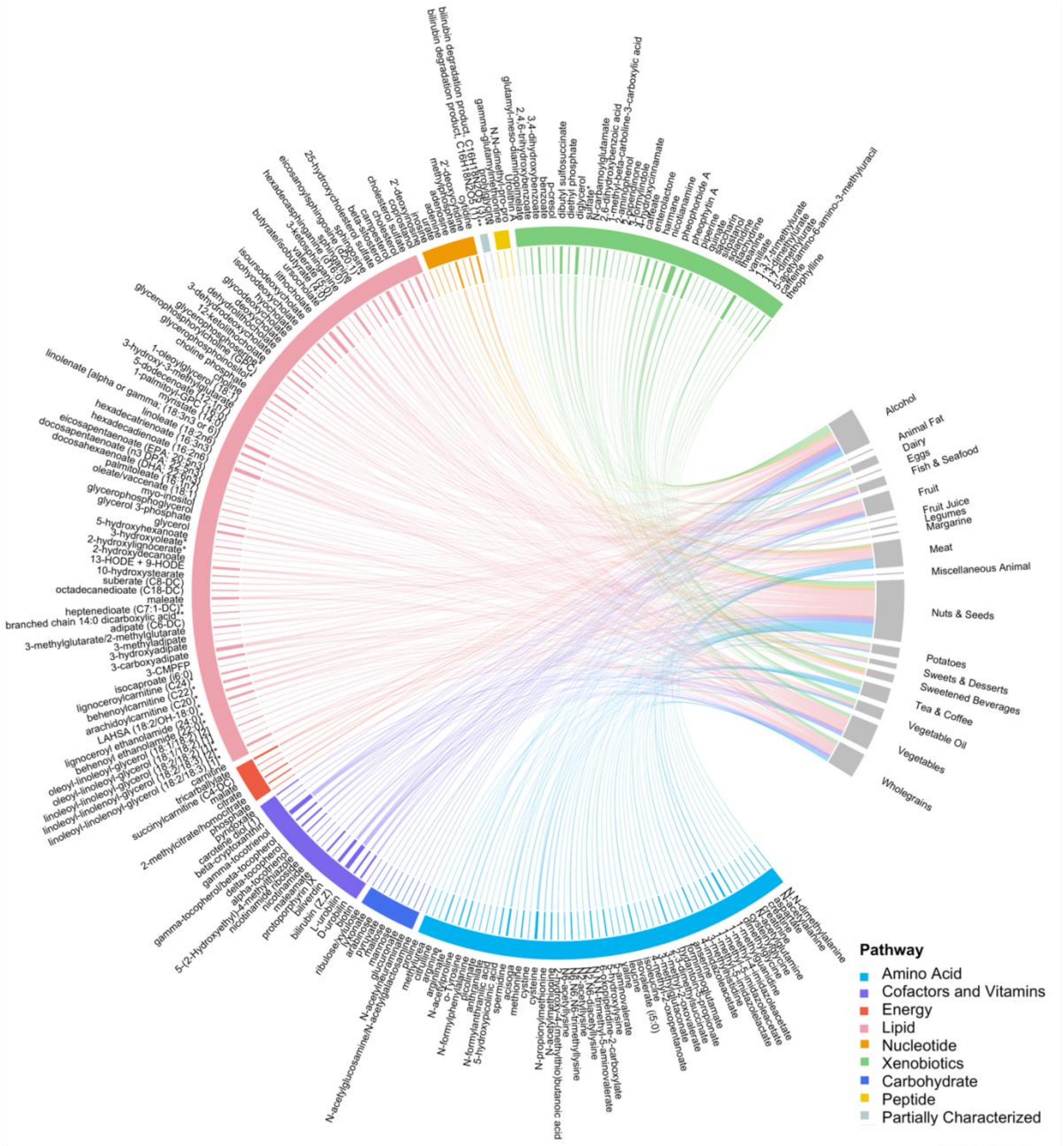

Supplementary Figure 4: Chord diagram of 411 significant positive and negative associations between characterised faecal metabolites and food and beverage groups. Associations identified from fixed effects meta-analysis of linear mixed effects regression models corrected for age, sex, BMI, and twin family structure in the TwinsUK (n=1,810) and ZOE PREDICT1 (n=318) cohorts, were considered significant below a Bonferroni-derived threshold of 9.78×10^{-5}. Faecal metabolites are organised and coloured according to their metabolic super pathways.

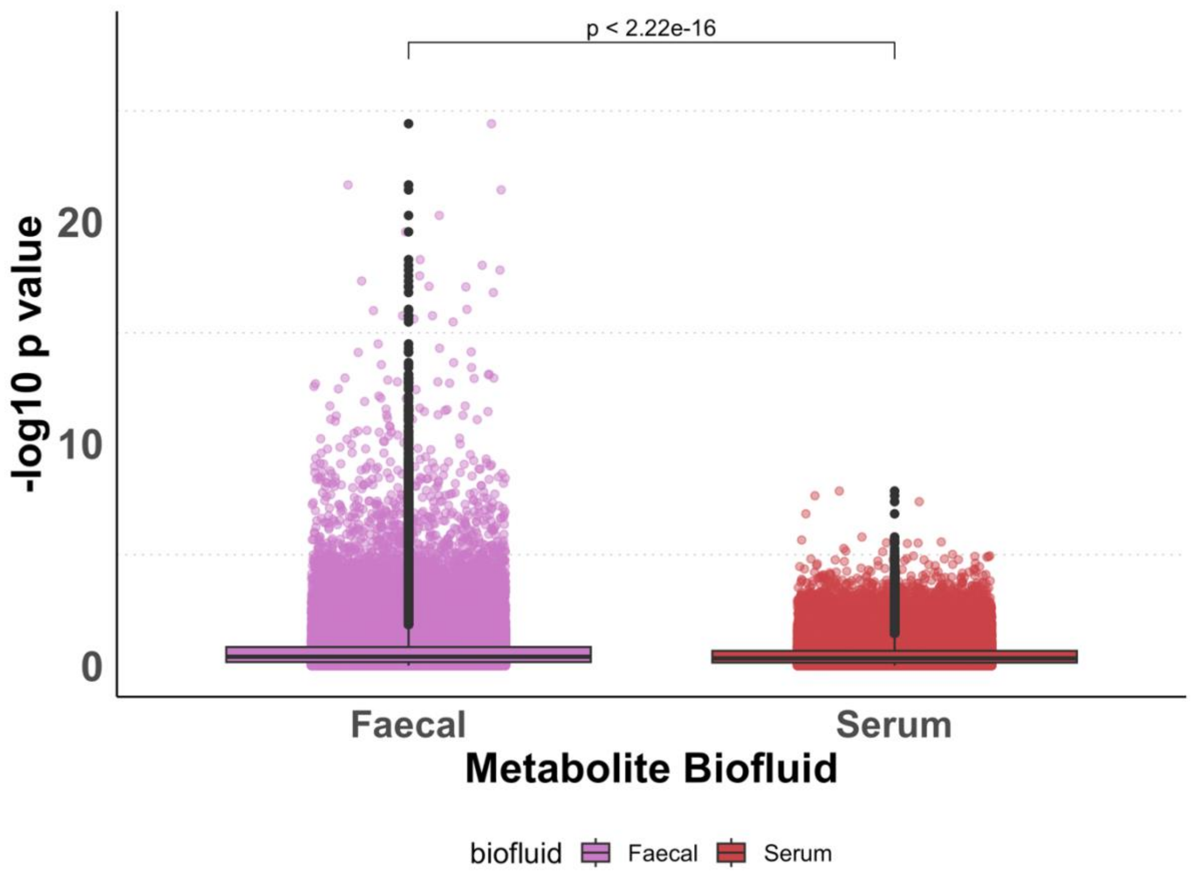

Supplementary Figure 5: Associations between dietary associated faecal or serum metabolites with gut microbial species for 657 participants from TwinsUK with faecal, serum and gut metagenomics data. Linear mixed effects regression models adjusted for age, sex, BMI and twin family structure were used. The Wilcoxon rank-sum test was used to compare association strengths, quantified using the -$\log_{10}$ of the p values, between faecal or serum metabolites and gut microbial species.

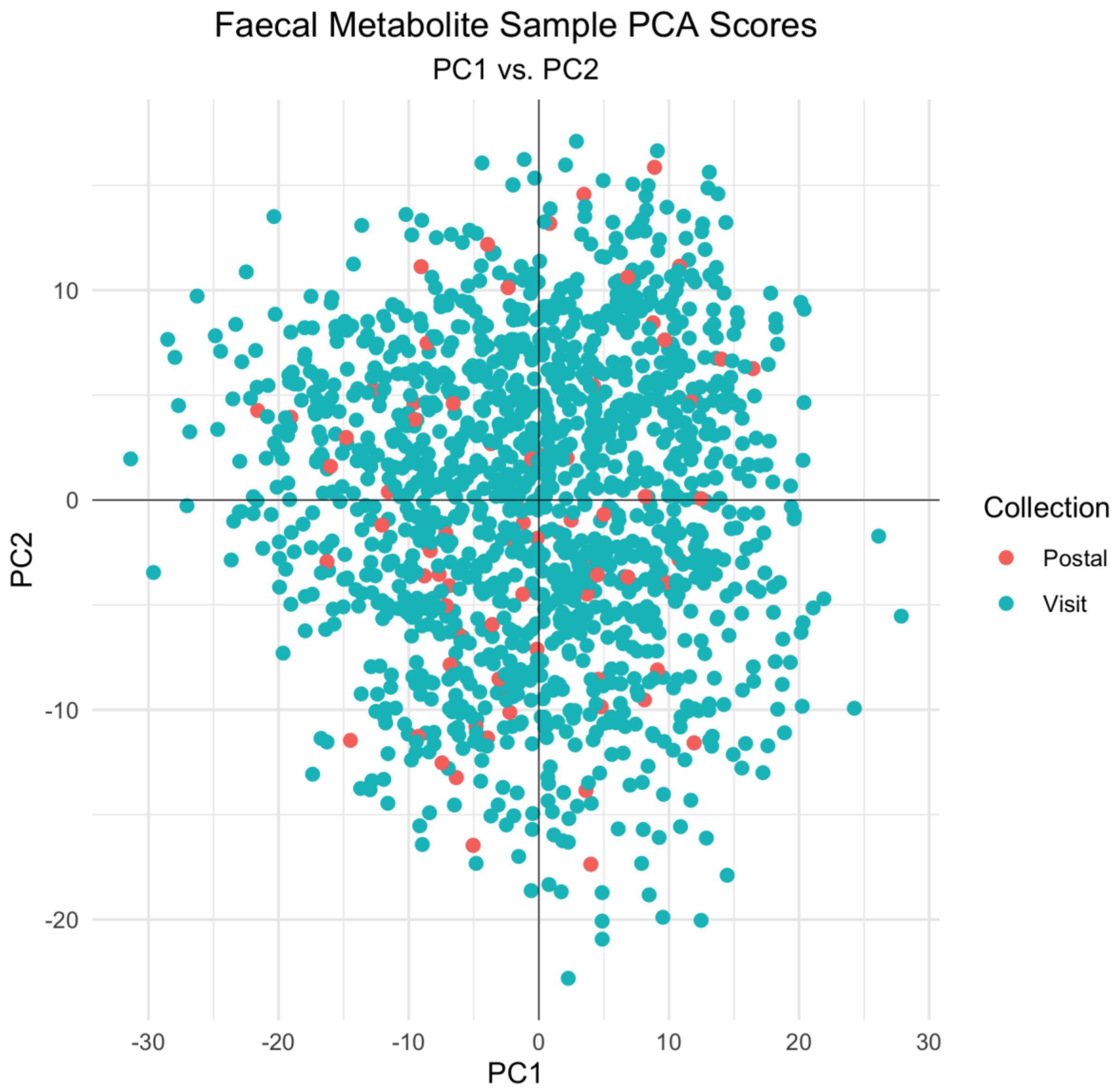

Supplementary Figure 6: PCA of faecal samples collected at a TwinsUK clinic visit or returned via post. Stool samples collected at home were refrigerated for up to two days prior to the clinical visit or sent as soon as possible via post using blue Royal Mail safe boxes to ensure the samples were transported under the correct cooled temperature.

Supplementary Tables

Supplementary Table 1: Amalgamated food and beverage groups for the calculation of the Plant-based Diet Index (PDI) and its healthful (hPDI) and unhealthful (uPDI) derivation. FFQ line items were grouped according to the PDI food group definitions by Satija et al. (2017)[1].

PDI Food Group	FFQ Line-Item Name	Food Group Scoring		
**Healthy**		**PDI**	**hPDI**	**uPDI**
Fruit	Apples, bananas, dried fruit, grapefruit, grapes, melons, oranges, peaches, pears, strawberries, tinned fruit	+	+	-
Legumes	Beans, lentils, tofu	+	+	-
Nuts & Seeds	Nuts salted, nuts unsalted, peanut butter, seeds	+	+	-
Tea & coffee	Coffee, decaf coffee, fruit tea, green tea, tea	+	+	-
Vegetable Oils	French dressing, other dressing, olive oil spread	+	+	-
Vegetables	Avocado, beansprouts, beetroot, broccoli, cabbage, carrots, cauliflower, coleslaw, garlic, green beans, green salad, ketchup, leeks, marmite, marrow, mushrooms, onions, parsnips, peas, peppers, pickles, sauces, spinach, sprouts, sweetcorn, tomatoes, vegetable soup, watercress	+	+	-
Wholegrain	Brown bread, brown rice, high fibre cereal, porridge, wholemeal bread, wholemeal pasta	+	+	-
**Less Healthy**		**PDI**	**hPDI**	**uPDI**
Fruit Juice	Fruit juice, smoothies	+	-	+
Refined Grains	Crackers, crispbread, cornflakes/rice Krispies, muesli, naan/poppadom/tortillas, white bread, white pasta, white rice	+	-	+
Potatoes	Boiled potatoes, chips/roast potatoes, crisps, potato salad	+	-	+
Sweetened Beverages	Cocoa, coffee whitener, fizzy drinks, fruit squash, Horlicks, low fat hot chocolate, low calorie fizzy drinks	+	-	+
Sweets & Desserts	Cereal bars, chocolate bars, chocolate biscuits, dark chocolate, homemade buns, homemade cake, homemade fruit pies, homemade sponge, jam, low fat biscuits, milk/white chocolate, plain biscuit, readymade buns, readymade cake, readymade fruit pies, readymade sponge, sugar, sugar topped cereal, sweets	+	-	+
**Animal Foods**		**PDI**	**hPDI**	**uPDI**
Animal Fat	Butter, reduced fat butter	-	-	-
Dairy	Cheese, cottage cheese, dairy dessert, double cream, full fat yoghurt, ice cream, low fat yoghurt, milk, milk puddings, reduced fat cheese, single cream	-	-	-
Eggs	Eggs	-	-	-
Fish & Seafood	Fish fingers, fried fish, oily fish, roe, shellfish, white fish	-	-	-
Meat	Bacon, beef, burger, chicken, corned beef, ham, lamb, lasagne, liver, meat soup, pork, sausages	-	-	-
Miscellaneous Animal-based Products	Low calorie salad cream, pizza, quiche, salad cream, savoury pies	-	-	-
**Excluded**		**PDI**	**hPDI**	**uPDI**
Margarine	Cholesterol reducing spread, hard margarine, low-fat spread, other margarine, polyunsaturated margarine, very low-fat spread			
Alcohol	Beer, port, red wine, spirits, white wine			

Supplementary Table 2: FFQ derived Alternate Mediterranean Diet Score (aMED) food and nutrient groups and their population medians. Amalgamated FFQ derived food or nutrient groups used to calculate the aMED score according to the modifications described by Fung et al. (2005)[2].

aMED Food or Nutrient Group	FFQ Line-Item or Nutrient Name	Population Median
Vegetables	Avocado, beansprouts, beetroot, broccoli, cabbage, carrots, cauliflower, garlic, green salad, leeks, marrow, mushrooms, onions, parsnips, peppers, spinach, sprouts, sweetcorn, tomatoes, vegetable soup, watercress	233.3 g d^{-1}
Fruit	Apples, bananas, dried fruit, fruit juice, grapefruit, grapes, melon, oranges, peaches, pears, tinned fruit	225.0 g d^{-1}
Wholegrain	Brown bread, brown rice, cereal high fibre, crispbread, muesli, porridge, wholemeal bread, wholemeal pasta	101.9 g d^{-1}
Nuts	Nuts salted, nuts unsalted, peanut butter	6.9 g d^{-1}
Meat	Bacon, beef, burger, corned beef, ham, lamb, lasagne, liver, meat soup, pork, sausages, savoury pies	69.5 g d^{-1}
Legumes	Beans, green beans, lentils, peas	57.2 g d^{-1}
Fish & Seafood	Fish fingers, fried fish, oily fish, roe, shellfish, whitefish	39.6 g d^{-1}
Fatty Acid Ratio	Ratio of MUFA / SFA calculated from the FETA processed FFQ	1.0
Alcohol	Alcohol calculated from the FETA processed FFQ<br>Females: 5-25g d^{-1}<br>Males: 10-50g d^{-1}	Females: 4.1g d^{-1}<br>Males: 9.9g d^{-1}
**Excluded**		
FFQ Line Items	Boiled potatoes, butter, cereal, cereal bars, cheese, chicken, chips/roast pots, chocolate bars, chocolate biscuit, cholesterol spread, cocoa, coffee, coffee whitener, coleslaw, cottage cheese, crackers, crisps, dairy dessert, dark chocolate, decaf coffee, double cream, eggs, fizzy drinks, French dressing, fruit squash, fruit tea, full fat yoghurt, green tea, hard margarine, homemade buns, homemade cakes, homemade fruit pies, homemade sponge, Horlicks, ice cream, jam, ketchup, low calorie fizzy drinks, low calorie salad cream, low fat biscuit, low fat cocoa, low fat spread, low fat yoghurt, marmite, milk / white chocolate, milk puddings, naan/poppadom/tortillas, olive oil spread, other dressing, other margarine, pickles, pizza, plain biscuit, potato salad, polyunsaturated margarine, quiche, readymade buns, readymade cakes, readymade fruit pies, readymade sponge, reduced fat butter, reduced fat cheese, salad cream, sauces, seeds, single cream, smoothies, strawberries, sugar, sugar topped cereal, sweets, tea, tofu, very low-fat spread, white bread, white pasta, white rice	

Supplementary Table 3: FFQ derived Dietary Approaches to Stop Hypertension index (DASH) food or nutrient groups and their scoring. Amalgamated food or nutrient groups derived from the FFQ line items for the calculation of the DASH diet score according to Fung et al. (2008)[3].

DASH Food or Nutrient Group	Item Name	Group Scoring
Vegetables	Avocado, beansprouts, beetroot, broccoli, cabbage, carrots, cauliflower, coleslaw, garlic, green salad, leeks, marrow, mushrooms, onions, parsnips, peppers, pickles, sauces, spinach, sprouts, sweetcorn, tomatoes, vegetable soup, watercress	+
Fruit	Apples, bananas, dried fruit, fruit juice, grapefruit, grapes, melons, oranges, peaches, pears, smoothies, strawberries, tinned fruit	+
Nuts & Legumes	Beans, green beans, lentils, nuts salted, nuts unsalted, peanut butter, peas, seeds, tofu	+
Wholegrain	Brown bread, brown rice, high fibre cereal, porridge, wholemeal bread, wholemeal pasta	+
Low-fat Dairy	Cottage cheese, low-fat yoghurt, reduced fat cheese	+
Sweetened Beverages	Cocoa, coffee whitener, fizzy drinks, fruit squash, Horlicks, low-fat hot chocolate, low calorie fizzy drinks,	-
Meat	Bacon, beef, burger, corned beef, ham, lamb, lasagne, liver, meat soup, pork, sausages	-
Sodium	Sum of Sodium calculated from the FETA processed FFQ	-
**Excluded**		
FFQ Line Items	Beer, boiled potatoes, butter, cereal, cereal bars, cheese, chicken, chips/roast pots, chocolate bars, chocolate biscuit, cholesterol spread, coffee, crackers, crispbread, crisps, dairy dessert, dark chocolate, decaf coffee, double cream, eggs, fish fingers, French dressing, fried fish, fruit tea, full fat yoghurt, green tea, hard margarine, homemade buns, homemade cakes, homemade fruit pies, homemade sponge, ice cream, jam, ketchup, low calorie salad cream, low fat biscuit, low fat spread, marmite, milk / white chocolate, milk puddings, muesli, naan/poppadom/tortillas, oily fish, olive oil spread, other dressing, other margarine, pizza, plain biscuit, polyunsaturated margarine, port, potato salad, quiche, readymade buns, readymade cakes, readymade fruit pies, readymade sponge, red wine, reduced fat butter, roe, salad cream, savoury pies, shellfish, single cream, spirits, sugar, sugar topped cereal, sweets, tea, very low-fat spread, white bread, white fish, white pasta, white rice, white wine	

Supplementary Table 4: FFQ derived food groups for Total Meat % and Total Plant % indices. Amalgamated food groups from the FFQ line items for the calculation of the percentage of the diet constituted by plant-based foods or meat-based products. Groupings based on those defined for the PDI.

Food Group Name	FFQ Line-Item Name
Total Plant	Apples, avocado, bananas, beans, beansprouts, beetroot, boiled potatoes, broccoli, brown bread, brown rice, cabbage, carrots, cauliflower, cereal bars, chips/roast potatoes, chocolate bars, chocolate biscuits, cocoa, coffee, coffee whitener, coleslaw, cornflakes/rice Krispies, crackers, crispbread, crisps, dark chocolate, decaf coffee, dried fruit, fizzy drinks, French dressing, fruit juice, fruit squash, fruit tea, garlic, grapefruit, grapes, green beans, green salad, green tea, high fibre cereal, homemade buns, homemade cake, homemade fruit pies, homemade sponge, Horlicks, jam, ketchup, leeks, lentils, low calorie fizzy drinks, low fat biscuits, low fat hot chocolate, marmite, marrow, melons, milk/white chocolate, muesli, mushrooms, naan/poppadom/tortillas, nuts salted, nuts unsalted, olive oil spread, onions, oranges, other dressing, parsnips, peaches, peanut butter, pears, peas, peppers, pickles, plain biscuit, porridge, potato salad, readymade buns, readymade cake, readymade fruit pies, readymade sponge, sauces, seeds, smoothies, spinach, sprouts, strawberries, sugar, sugar topped cereal, sweetcorn, sweets, tea, tinned fruit, tofu, tomatoes, vegetable soup, watercress, white bread, white pasta, white rice, wholemeal bread, wholemeal pasta
Total Meat	Bacon, beef, burger, chicken, corned beef, ham, lamb, lasagne, liver, meat soup, pizza, pork, sausages, savoury pies

Supplementary Table 5: Coefficients for the calculation of the 10-year risk of a first hard atherosclerotic cardiovascular disease (ASCVD) event. Sex and race specific coefficients and equations used for the computation of 10-year risk of a first hard ASCVD event. Coefficients and equations derived from the ACC/AHA guidelines by Goff et al. (2013)[4]. The 10-year ASCVD risk score was only calculated for participants who were younger than 80 years old and had all relevant measurements taken within three years of their faecal sample (TwinsUK: n=1,720; ZOE PREDICT1: n=110). All participants in this subset self-reported as being of European ancestry.

Factor	Coefficient (Sex: Race)	
**Women**	**Coefficients (Female: White)**	**Equation**
Log Age (yr)	–29.799	Risk Score =<br>(-29.799 * Ln(Age)) +<br>(4.884 * Ln(Age) * Ln(Age)) +<br>(13.54 * Ln(Total Cholesterol mg/dL)) –<br>(3.114 * Ln(Age) * Ln(Total Cholesterol)) –<br>(13.578 * Ln(HDL mg/dL)) +<br>(3.149 * Ln(Age) * Ln(HDL)) +<br>(Treated * 2.019 * Ln(SBP mmHg)) +<br>(Untreated * 1.957 * Ln(SBP mmHg)) +<br>(7.574 * Current Smoker) –<br>(1.665 * Ln(Age) * Current Smoker) +<br>(0.661 x Diabetes) –<br>-29.18<br><br>10 year ASCVD Risk (%) =<br>(1 - (0.9665^(exp(Risk Score))) * 100
Log Age Squared	4.884	
Log Total Cholesterol (mg/dL)	13.54	
Log Age * Log Total Cholesterol	–3.114	
Log HDL Cholesterol (mg/dL)	–13.578	
Log Age * Log HDL Cholesterol	3.149	
Log Treated SBP (mmHg)	2.019	
Log Untreated SBP (mmHg)	1.957	
Current Smoker (1=Yes, 0=No)	7.574	
Log Age * Current Smoker	–1.665	
Diabetes (1=Yes, 0=No)	0.661	
Mean (Coefficient * Value)	-29.18	
Baseline Survival	0.9665	
**Men**	**Coefficients (Male: White)**	**Equation**
Log Age (yr)	12.344	Risk Score =<br>(12.344 * Ln(Age)) +<br>(11.85 * Ln(Total Cholesterol mg/dL)) -<br>(2.664 * Ln(Age) * Ln(Total Cholesterol)) -<br>(7.990 * Ln(HDL mg/dL)) +<br>(1.769 * Ln(Age) * Ln(HDL)) +<br>(Treated * 1.797 * Ln(SBP mmHg)) +<br>(Untreated * 1.764 * Ln(SBP mmHg)) +<br>(7.837 * Current Smoker) –<br>(1.795 * Ln(Age) * Current Smoker) +<br>(0.658 * Diabetes) -<br>61.18<br><br>10 year ASCVD Risk (%) =<br>(1 - (0.9144^(exp(Risk Score))) * 100
Log Total Cholesterol (mg/dL)	11.853	
Log Age * Log Total Cholesterol	–2.664	
Log HDL Cholesterol (mg/dL)	–7.990	
Log Age * Log HDL Cholesterol	1.769	
Log Treated Systolic BP (mmHg)	1.797	
Log Untreated Systolic BP (mmHg)	1.764	
Current Smoker (1=Yes, 0=No)	7.837	
Log Age * Current Smoker	–1.795	
Diabetes (1=Yes, 0=No)	0.658	
Mean (Coefficient×Value)	61.18	
Baseline Survival	0.9144	

References

1. Satija, A. *et al.* Healthful and unhealthful plant-based diets and the risk of coronary heart disease in US adults. *J. Am. Coll. Cardiol.* **70**, 411–422 (2017).
2. Fung, T. T. *et al.* Diet-quality scores and plasma concentrations of markers of inflammation and endothelial dysfunction. *Am. J. Clin. Nutr.* **82**, 163–173 (2005).
3. Fung, T. T. *et al.* Adherence to a DASH-Style Diet and Risk of Coronary Heart Disease and Stroke in Women. *Arch. Intern. Med.* **168**, 713–720 (2008).
4. Goff, D. C. *et al.* 2013 ACC/AHA Guideline on the Assessment of Cardiovascular Risk: A Report of the American College of Cardiology/American Heart Association Task Force on Practice Guidelines. *Circulation* **129**, S49-73 (2014).
