# Supplementary material for: Faecal metabolites as a readout of habitual diet capture dietary interactions with the gut microbiome
Source: Nat Commun. 2025 Dec 4;16:10051. doi: 10.1038/s41467-025-66046-7 (PMC12678775; doi:10.1038/s41467-025-66046-7)
Supplement: Supplementary file 2 — Description of Additional Supplementary Files [file 41467_2025_66046_MOESM2_ESM.pdf]

Description of Additional Supplementary Files

File Name: Supplementary Data 1

Description: Random Forest binary classification model input features, hyperparameters, and evaluation metrics for dietary indices, food and beverage groups and 10-year atherosclerotic cardiovascular disease (ASCVD) risk. Two types of models were defined, the *Null* models which included only the covariates (age, sex, BMI) and the *Metabolite* models which included faecal metabolites and covariates identified as important predictors using the Boruta algorithm. Models were trained using an 80% partition of the TwinsUK dataset and tested with the remaining 20% hold out set. Trained models were tested using the ZOE PREDICT1 validation cohort. Differences in AUC scores were assessed using DeLong's test and the resulting p values were considered significant if they were below the Bonferroni thresholds.

File Name: Supplementary Data 2

Description: Random Forest regression model input features, hyperparameters, and evaluation metrics for dietary indices, food and beverage groups and 10-year ASCVD risk. Two types of models were defined, the *Null* models which included only the covariates (age, sex, BMI) and the *Metabolite* models which included faecal metabolites and covariates identified as important predictors using the Boruta algorithm. Models were trained using an 80% partition of the TwinsUK dataset and tested with the remaining 20% hold out set. Trained models were tested using the ZOE PREDICT1 validation cohort.

File Name: Supplementary Data 3

Description: Permutation feature importance for Random Forest binary classification and regression models. Input features were initially selected using the Boruta algorithm and feature importance was computed using the permutation variable importance approach implemented using the R package, ranger (v.0.16.0).

File Name: Supplementary Data 4

Description: Dietary index and food and beverage group Random Forest classification model predictions with reduced input features for models with an original area under the curve (AUC) > 0.70. The number of input features was chosen based on a recursive feature elimination approach (Methods). Input feature importance was calculated by means of permutation feature importance.

File Name: Supplementary Data 5

Description: Dietary index and food and beverage group metabolite panels consisting of a total of 54 unique metabolites. A value of 1 in the columns 'Diet Index' and 'Food Group' indicates the presence of the faecal metabolite in either the Diet Index or Food Group panel.

File Name: Supplementary Data 6

Description: The Retention Index (RI), quant m/z and tentative details for the eleven compounds of unknown identity included in the panel of 54 dietary predictive metabolites. Tentative details describe any information thought to be known about the compounds. The RI is a normalised measure of chromatographic retention, calculated from a compound's elution time relative to surrounding retention markers, correcting for retention time drift across runs. Data provided by Metabolon (inc).

File Name: Supplementary Data 7

Description: Associations of 526 characterised faecal metabolites with habitual intakes of 20 food and beverage groups, with intakes adjusted using the residual energy method. Associations were identified using two-sided linear mixed-effects regression (LMER) models in the TwinsUK (n=1,810) and ZOE PREDICT1 (n=318) cohorts, adjusting for age, sex, BMI, and twin family structure. Results from both cohorts were combined using a fixed-effects meta-analysis. Associations were considered significant if the estimated effect sizes (β) were in the same direction across both cohorts and the meta-analysis p value was below the Bonferroni-corrected threshold ($p < 9.78 \times 10^{-5}$). Statistics reported include β estimates, 95% confidence intervals (CI), standard errors (SE), and exact p values from the fixed effects meta-analysis, as well as cohort-specific β, 95% CI, SE, and p values for TwinsUK and ZOE PREDICT1 individually. Significant associations are highlighted in green.

File Name: Supplementary Data 8

Description: 414 significant associations between 211 characterised faecal metabolites and habitual intakes of 19 food and beverage groups. Metabolites that were uniquely associated with one just one food group are labelled as '*Unique*', whereas metabolites associated with multiple food groups are labelled as '*Multiple*'. Associations were considered significant if the estimated effect sizes (β) were in the same direction across both cohorts and the meta-analysis p value was below the Bonferroni-corrected threshold ($p < 9.78 \times 10^{-5}$). Statistics reported include β estimates, 95% confidence intervals (CI), standard errors (SE), and exact p values from the fixed effects meta-analysis.

File Name: Supplementary Data 9

Description: Associations of 211 dietary associated faecal metabolites with microbiome alpha diversity (Sahnnon Index) in the TwinsUK (n=726) and ZOE PREDICT 1 (n=318) cohorts. Associations were identified using two-sided linear mixed-effects regression (LMER) models in each cohort, adjusting for age, sex, BMI, and twin family structure. Results were combined using fixed-effects meta-analysis. The column "*Beta Same Direction*" indicates whether the direction of association was consistent across both cohorts. Associations were considered significant if the effect size (β) was in the same direction across cohorts and the meta-analysis p value was below the Bonferroni threshold ($p < 2.51 \times 10^{-4}$). Statistics reported include β estimates, 95% confidence intervals (CI), standard errors (SE), and exact p values from the fixed effects meta-analysis, as well as cohort-specific β, 95% CI, SE, and p values for TwinsUK and ZOE PREDICT1 individually. Significant associations are highlighted in green.

File Name: Supplementary Data 10

Description: Permutational multivariate analysis of variance (PERMANOVA) results of the impact of 211 dietary associated faecal metabolites on intra-individual microbiome community structure (Bray-Curtis dissimilarity) in the TwinsUK (n=474) and ZOE PREDICT1 (n=219) cohorts. Analyses were performed using PERMANOVA with 10,000 permutations, adjusting for age, sex, and BMI. To account for non-independence of observations, one twin per pair was removed from twin pairs present in both cohorts. Weighted R^2 values were calculated from the cohort-specific R^2 values and sample sizes. Associations were considered significant if the permutation-derived p value was below the Bonferroni threshold derived for each cohort (TwinsUK: $p < 2.51 \times 10^{-4}$; ZOE PREDICT1: $p < 2.53 \times 10^{-4}$). Reported statistics include the pseudo-F statistic, R^2, weighted R^2, and permutation p values. Significant associations are highlighted in green.

File Name: Supplementary Data 11

Description: Associations between food and beverage groups with gut microbial species relative abundances for the TwinsUK and ZOE PREDICT 1 cohorts (sample sizes for each pairwise combination indicated in the columns "*N*"). Associations were identified using two-sided linear mixed-effects regression (LMER) models in each cohort, adjusting for age, sex, BMI, and twin family structure. Results were combined using fixed-effects meta-analysis. Associations were considered significant if the effect size (β) was in the same direction across both cohorts and the meta-analysis false discovery rate (FDR, Benjamini–Hochberg) was below 0.1. Statistics reported include β estimates, 95% confidence intervals (CI), standard errors (SE), p values and FDR corrected p values from the fixed effects meta-analysis, as well

as cohort-specific β, 95% CI, SE, and p values for TwinsUK and ZOE PREDICT1 individually. Significant associations are highlighted in green.

File Name: Supplementary Data 12

Description: Associations of 211 dietary associated faecal metabolites with gut microbial species relative abundances for the TwinsUK and ZOE PREDICT 1 cohorts (sample sizes for each pairwise combination indicated in the columns "*N*"). Associations were identified using two-sided linear mixed-effects regression (LMER) models in each cohort, adjusting for age, sex, BMI, and twin family structure. Results were combined using a fixed-effects meta-analysis. Associations were considered significant if the effect size (β) was in the same direction across both cohorts and the meta-analysis p value was below the Bonferroni threshold defined in the TwinsUK cohort ($p < 6.67\times10^{-5}$). Statistics reported include β estimates, 95% confidence intervals (CI), standard errors (SE), and p values from the fixed effects meta-analysis, as well as cohort-specific β, 95% CI, SE, and p values for TwinsUK and ZOE PREDICT1 individually. Significant associations are highlighted in green.

File Name: Supplementary Data 13

Description: Results from causal mediation analyses for both metabolites and species as mediators in the TwinsUK cohort (n=726). Only trios of associations (food group–species–metabolite) where all three pairwise associations passed multiple testing correction were included. Linear mixed-effects regression (LMER) models (two-sided) were used for direct and indirect paths, adjusting for age, sex, BMI, and twin family structure. The output from the LMER models for food groups, microbial species abundances, and faecal metabolites were used as the input to the mediation models. Reported statistics include regression coefficients (β) with 95% confidence intervals (CI), standard errors (SE) and p values for each model. The average causal mediation effect (ACME) and average direct effect (ADE) are reported, with their sum representing the total effect. The proportion mediated represents the percentage of the total effect attributable to the mediator. Mediatory effects were considered significant at $p < 0.05$.

File Name: Supplementary Data 14

Description: Results from the testing of serum and faecal metabolites to predict adherence to dietary patterns and high and low habitual consumption of food & beverage groups. A subset of TwinsUK with serum metabolomics data (n=1,618) were used. This subset was split into 80% train and 20% test sets. The training set was used to select important faecal or serum predictors with the Boruta algorithm and then train Random Forest binary classification models. Trained and optimised models were tested using the 20% test set. The AUC scores

for serum and faecal metabolite models were compared using a two-sided DeLong's test to assess whether there was any significant difference between the models' AUCs.
