# Supplementary material for: Faecal metabolites as a readout of habitual diet capture dietary interactions with the gut microbiome
Source: Nat Commun. 2025 Dec 4;16:10051. doi: 10.1038/s41467-025-66046-7 (PMC12678775; doi:10.1038/s41467-025-66046-7)
Supplement: Supplementary file 17 — Reporting Summary [file 41467_2025_66046_MOESM17_ESM.pdf]

nature portfolio

Corresponding author(s): Mario Falchi

Last updated by author(s): Oct 21, 2025

Reporting Summary

Nature Portfolio wishes to improve the reproducibility of the work that we publish. This form provides structure for consistency and transparency in reporting. For further information on Nature Portfolio policies, see our Editorial Policies and the Editorial Policy Checklist.

Statistics

For all statistical analyses, confirm that the following items are present in the figure legend, table legend, main text, or Methods section.

n/a	Confirmed	
☐	☒	The exact sample size (*n*) for each experimental group/condition, given as a discrete number and unit of measurement
☐	☒	A statement on whether measurements were taken from distinct samples or whether the same sample was measured repeatedly
☐	☒	The statistical test(s) used AND whether they are one- or two-sided<br>*Only common tests should be described solely by name; describe more complex techniques in the Methods section.*
☐	☒	A description of all covariates tested
☐	☒	A description of any assumptions or corrections, such as tests of normality and adjustment for multiple comparisons
☐	☒	A full description of the statistical parameters including central tendency (e.g. means) or other basic estimates (e.g. regression coefficient) AND variation (e.g. standard deviation) or associated estimates of uncertainty (e.g. confidence intervals)
☐	☒	For null hypothesis testing, the test statistic (e.g. *F*, *t*, *r*) with confidence intervals, effect sizes, degrees of freedom and *P* value noted<br>*Give P values as exact values whenever suitable.*
☒	☐	For Bayesian analysis, information on the choice of priors and Markov chain Monte Carlo settings
☐	☒	For hierarchical and complex designs, identification of the appropriate level for tests and full reporting of outcomes
☐	☒	Estimates of effect sizes (e.g. Cohen's *d*, Pearson's *r*), indicating how they were calculated

Our web collection on statistics for biologists contains articles on many of the points above.

Software and code

Policy information about availability of computer code

Data collection	No software was used to collect the dietary, metabolomics or metagenomics data used in this study.

Data analysis

Food frequency questionnaire data were processed with the FETA software (v. 2.53) (https://www.mrc-epid.cam.ac.uk/research/measurement-platform/dietary-assessment/feta/). Metagenome data was processed with the YAMP pipeline (v.0.9.5.0, mode "qc") with default parameters (https://github.com/alesssia/YAMP). Taxonomic profiling was carried out using MetaPhlAn (v.4.beta.2), with the January 2021 species-specific database (https://github.com/biobakery/MetaPhlAn). All analyses were carried out using R version (v.4.3.2) using open source libraries including:
vegan (v.2.6.4)
dbscan (v.1.2.0)
microbiome (v.1.24.0)
ranger (v.0.16.0)
Boruta (v.8.0.0)
caret (v.6.0.94)
pROC (v.1.18.5)
lme4 (v.1.1-33)
lmerTest (v.3.1.3)
metafor (v.4.4-0)
ComplexHeatmap (v.2.18.0)
mediation (v.4.5.0)

For manuscripts utilizing custom algorithms or software that are central to the research but not yet described in published literature, software must be made available to editors and reviewers. We strongly encourage code deposition in a community repository (e.g. GitHub). See the Nature Portfolio guidelines for submitting code & software for further information.

Data

Policy information about availability of data

All manuscripts must include a data availability statement. This statement should provide the following information, where applicable:

- Accession codes, unique identifiers, or web links for publicly available datasets
- A description of any restrictions on data availability
- For clinical datasets or third party data, please ensure that the statement adheres to our policy

The raw metagenomic sequence data used in this study have been deposited in the European Bioinformatics Institute European Nucleotide Archive database (TwinsUK accession code: PRJEB98467; ZOE PREDICT1 accession code: PRJEB39223). All data relating to TwinsUK samples have been deposited to the TwinsUK BioResource data management team. These data and non-metagenomic data for ZOE PREDICT1 are available by application to the Twin Research Executive Access committee (TREC) at King's College London. The TwinsUK BioResource is managed by TREC, which provides governance of access to TwinsUK data and samples. TwinsUK data users are bound by data sharing agreement set out in the data access application form, which includes responsibilities with respect to third party data sharing and maintaining participant privacy. Further responsibilities include a responsibility to acknowledge data sharing.

Research involving human participants, their data, or biological material

Policy information about studies with human participants or human data. See also policy information about sex, gender (identity/presentation), and sexual orientation and race, ethnicity and racism.

Reporting on sex and gender

Participants included from the TwinsUK and ZOE PREDICT 1 cohorts were predominantly female (86.0% and 74.3%, respectively). Reported sex was determined by sex assigned at birth. Sex was included as a predictor for all null machine learning models as well as when sex was identified as an important predictor during feature selection. Sex was not included as an input variable for the prediction of 10-year ASCVD risk as to avoid data leakage due to the inclusion of sex in risk score computation. All statistical tests were corrected for sex which was coded as a factor variable with two levels, 0 for females or 1 for males.

Reporting on race, ethnicity, or other socially relevant groupings

All participants of the TwinsUK sample and >91% of PREDICT 1 self reported being of European ancestry.

Population characteristics

The descriptive characteristics and estimated daily macro nutrient intakes for the study populations are detailed in the Results in Table 1. Age and BMI were included as covariates in all analyses. 1,810 participants aged 18-92 years were included from the TwinsUK with BMIs ranging from 15.7-50.7 kg/m2. 837 participants were included from ZOE PREDICT 1, with ages ranging from 18-66 years and BMI from 17.0-53.2 kg/m2. The non-independence of twin observations was accounted for by maintaining twin pairs in the same fold for machine learning cross-validation hyperparamter optimisation or by including family relations as a random effect in linear mixed effects regression models.

Recruitment

TwinsUK is a national register of adult twins recruited as volunteers without selecting for any particular disease or trait. Volunteers provided informed consent, and the study was approved by the North West – Liverpool Central Research Ethics Committee (REC Ref: 19/NW/0187) via the Integrated Research Application System (IRAS 258513)
The ZOE PREDICT 1 cohort recruitment program is described at https://protocolexchange.researchsquare.com/article/pex-802/v1.

Ethics oversight

For TwinsUK, the study was approved by the North West – Liverpool Central Research Ethics Committee (REC Ref: 19/NW/0187) via the Integrated Research Application System (IRAS 258513). For the ZOE PREDICT1 study, ethical approval was obtained in the UK from the Research Ethics Committee via the Integrated Research Application System (IRAS 236407). All individuals provided informed consent, and the trial was registered on ClinicalTrials.gov (registration number: NCT03479866).

Note that full information on the approval of the study protocol must also be provided in the manuscript.

Field-specific reporting

Please select the one below that is the best fit for your research. If you are not sure, read the appropriate sections before making your selection.

- [x] Life sciences
- [ ] Behavioural & social sciences
- [ ] Ecological, evolutionary & environmental sciences

For a reference copy of the document with all sections, see nature.com/documents/nr-reporting-summary-flat.pdf

Life sciences study design

All studies must disclose on these points even when the disclosure is negative.

Sample size

In this study a total of 2,647 participants were considered (TwinsUK = 1,810; ZOE PREDICT 1 = 837). Sub-groups from each cohort were considered for each analyses based on the availability of habitual diet, faecal metabolite or gut metagenome data. The full breakdown is detailed in Figure 1.

For analyses that included dietary data and faecal metabolites, subjects were selected if habitual dietary data had been collected within 3 years of the faecal sample for TwinsUK (n=1,810). Dietary data was collected at the same time as faecal samples for PREDICT1 (n=318).
For analyses that included dietary data and gut metagenomics data, for TwinsUK 726 participants of the 1,810 individuals had available gut metagenomics data whereas from the ZOE PREDICT1 cohort, a total of 837 participants were included with gut metagenomics data (including the 318 with faecal metabolite data). These study populations were selected based on maximising sample size for each analysis.

Data exclusions

Upon final study population selection, participants were excluded based on either incomplete food frequency questionnaires (>10 line items left unanswered) or due to unrealistic habitual dietary reporting (ratio of total energy intake (kcal) to the participants basal metabolic rate was more than two standard deviations from the mean). For metagenomic analysis, ecologically abnormal samples were identified and excluded through density-based spatial clustering of the first two principal coordinates from principal coordinate analysis (PCoA) using Bray-Curtis dissimilarities.

Replication

Results from the linear mixed effects models with the same effect size direction in the discovery and replication cohorts were then meta-analysed using fixed effects meta-analysis as implemented via the R package metafor (v.4.4-0). Reported effect size and p values from the meta-analysis were considered replicated across both cohorts if p values from the meta-analysis were below the Bonferroni thresholds defined within the TwinsUK discovery cohort. For associations with gut microbial species, p values from the meta-analysis were considered replicated at FDR<0.1.

Randomization

N/A: This is an observational study. Habitual dietary data was collected through the use of food frequency questionnaires and investigated.

Blinding

N/A: As specified above, real dietary exposure was investigated as opposed to diet as treatment.

Reporting for specific materials, systems and methods

We require information from authors about some types of materials, experimental systems and methods used in many studies. Here, indicate whether each material, system or method listed is relevant to your study. If you are not sure if a list item applies to your research, read the appropriate section before selecting a response.

Materials & experimental systems

n/a	Involved in the study	
x		Antibodies
x		Eukaryotic cell lines
x		Palaeontology and archaeology
x		Animals and other organisms
	x	Clinical data
x		Dual use research of concern
x		Plants

Methods

n/a	Involved in the study	
x		ChIP-seq
x		Flow cytometry
x		MRI-based neuroimaging

Clinical data

Policy information about clinical studies
All manuscripts should comply with the ICMJE guidelines for publication of clinical research and a completed CONSORT checklist must be included with all submissions.

Clinical trial registration

NCT03479866

Study protocol

The full protocol for the ZOE PREDICT 1 study can be found at: https://protocolexchange.researchsquare.com/article/pex-802/v1

Data collection

For the ZOE PREDICT 1 cohort, data was collected at St Thomas' Hospital, London between 5th June 2018 and 8th May 2019.

Outcomes	N/A

Plants

Seed stocks	*Report on the source of all seed stocks or other plant material used. If applicable, state the seed stock centre and catalogue number. If plant specimens were collected from the field, describe the collection location, date and sampling procedures.*
Novel plant genotypes	*Describe the methods by which all novel plant genotypes were produced. This includes those generated by transgenic approaches, gene editing, chemical/radiation-based mutagenesis and hybridization. For transgenic lines, describe the transformation method, the number of independent lines analyzed and the generation upon which experiments were performed. For gene-edited lines, describe the editor used, the endogenous sequence targeted for editing, the targeting guide RNA sequence (if applicable) and how the editor was applied.*
Authentication	*Describe any authentication procedures for each seed stock used or novel genotype generated. Describe any experiments used to assess the effect of a mutation and, where applicable, how potential secondary effects (e.g. second site T-DNA insertions, mosiacism, off-target gene editing) were examined.*
